# A retrospective cohort study to evaluate disease burden, health care resource utilization, and costs in patients with breast cancer in Dubai, UAE

**DOI:** 10.1186/s12913-024-11193-8

**Published:** 2024-07-12

**Authors:** DMM Hamza, MWA Zayed, N Tahoun, M Farghaly, S Kumaresan, BC Ramachandrachar, A Ali

**Affiliations:** 1 Medical oncology, Dubai Academic Health Cooperation, Dubai, UAE; 2 Pfizer Inc. Ltd, Dubai, United Arab Emirates; 3 Health Economics & Insurance Policies Department, Dubai Health Authority, Dubai, UAE; 4 EMEA Consulting Services, IQVIA, Bangalore, India; 5 Real-World Evidence, IQVIA, 11th Floor, Convention Tower, DWTC, Al Saada Street Dubai, Dubai, United Arab Emirates

**Keywords:** Breast cancer, Disease burden, Health care resource utilization, E-claims data, Events of special interest, CDK4/6 inhibitors

## Abstract

**Background:**

The current study evaluated the disease burden, health care resource utilization and analyzed the cost burden due to events of special interest among patients with breast cancer (BC) diagnosed and treated in Dubai, United Arab Emirates (UAE), in general and in the subset of patients treated with cyclin-dependent kinase (CDK) 4/6 inhibitors.

**Methods:**

This retrospective cohort study, using insurance e-claims data from Dubai Real-World Database, was conducted from 01 January 2014 to 30 September 2021. Female patients aged ≥ 18 years with at least 1 diagnosis claim for BC and with continuous enrollment during the index period were included.

**Results:**

Overall, 8,031 patients were diagnosed with BC (median age: 49.0 years), with the majority (68.1%) being in 41–60-year age group. During the post-index period, BC-specific costs contributed to 84% of the overall disease burden among patients with BC. Inpatient costs (USD 16,956.2) and medication costs (USD 10,251.3) contributed significantly to BC-specific costs. In the subgroup of patients in whom CDK4/6 inhibitors were part of the treatment regimen (*n* = 174), CDK4/6 inhibitors were commonly prescribed in combination with aromatase inhibitors (41.4%) and estrogen receptor antagonists (17.9%). In patients with BC, health care costs due to events of special interest (*n* = 1,843) contributed to 17% of the overall disease cost burden.

**Conclusion:**

The study highlights the significant cost burden among patients with BC, with BC-specific costs contributing to 84% of the overall disease cost burden. Despite few limitations such as study population predominantly comprising of privately insured expatriate patients and only direct healthcare costs being assessed in the current study, most indicative costs have been captured in the study, by careful patient selection and cost comparisons, as applicable. The findings can guide key health care stakeholders (payers and providers) on future policy measures aiming to reduce the cost burden among patients with BC.

**Supplementary Information:**

The online version contains supplementary material available at 10.1186/s12913-024-11193-8.

## Background

Globally, breast cancer (BC) is the most frequently occurring cancer, with an estimated incidence of 12.5% [1]. It is also associated with a high mortality rate of ~ 7%, accounting for 1 in 6 cancer deaths among women [2]. According to United Arab Emirates (UAE) epidemiological data, BC accounts for a major diagnosis burden among non-communicable diseases, with 1,030 new cases (both sexes) in 2020, accounting for 21.4% of all cancers and 222 deaths [3, 4]. There are multiple BC subtypes based on genome sequencing and the presence or absence of molecular markers for estrogen or progesterone receptors and human epidermal growth factor 2 (ERBB2), also known as human epidermal growth factor receptor 2 (HER2), with the majority of BC tumors expressing estrogen receptors and/or progesterone receptor (PR) [5, 6].

The current treatment choice for BC is dependent on the stage and grade of disease, as well as the BC molecular subtype [7]. Depending on the BC subtype, personalized targeted treatment approaches are employed using endocrine therapy (selective estrogen receptor modulators, selective estrogen receptor degraders), aromatase inhibitors, anti-HER2 therapy (trastuzumab, pertuzumab, lapatinib, pyrotinib), and poly adenosine diphosphate (ADP)-ribose polymerase inhibitors (olaparib, talazoparib) [7, 8]. Additionally, the advent of novel agents, including phosphatidylinositol-3-kinase (PI3K)/Akt and mammalian target of rapamycin (mTOR) pathway inhibitors (buparlisib, everolimus, and pictilisib), CDK4/6 inhibitors (palbociclib, ribociclib and abemaciclib), antibody-drug conjugates (trastuzumab-deruxtecan), and immune checkpoint inhibitors (pembrolizumab) have revolutionized the BC treatment landscape [7–11].

Despite tremendous advancements in the management of BC, significant economic concerns persist, primarily attributable to long-term inpatient and outpatient care for both affected individuals and the health care system [12], . In developed nations such as the USA [13], the overall average cost per patient 2 years post-diagnosis of BC is United States dollars (USD) 103,735 [14]. In a cross-sectional study conducted in Iran, the average annual cost reported for each patient with BC was USD 11,979.09, with direct costs being the major contributor [15]. Similarly, in a retrospective cross-sectional, prevalence-based study conducted in the Kingdom of Saudi Arabia in 2018, the total estimated cost for treating BC was USD 13.345 million, with the average cost per patient varying across different BC stages, ranging from USD 14,249 in stage I to USD 81,489 USD in stage IV. Medication and hospitalization costs were the major cost drivers [12]. Cost-effectiveness analysis studies have emphasized therapeutic drug costs to be a major driving force behind the high economic burden [16, 17]. Studies have shown that treatment-related adverse events also contribute significantly to the cost burden [18].

Cancer care in the UAE has undergone significant evolution in recent years, with approximately 20 cancer care facilities, including 4 comprehensive cancer centers, now operational. Furthermore, notable advancements in cancer screening programs specific to certain regions have also been observed, e.g., the Dubai Health Authority cancer screening program, ITMENAN, WEQAYA, and IFHAS. specifically for citizens of UAE and BASMAH, for non-citizens of UAE. Recently, robotic surgery, artificial intelligence, and precision oncology have gained momentum in cancer care in the UAE. The Emirates Medical Association has approved the development of a palliative and supportive care working group to create public awareness about palliative care. The UAE government covers the cost of cancer treatment for UAE nationals, while the expat population is covered by private sectors. Cancer care funded by the government is also available outside the UAE in instances where treatment is unavailable within the UAE. Nevertheless, to achieve more comprehensive, high-quality cancer care in the UAE, there is a need for implementation of following measures; a national screening program for the UAE; a national cancer control plan to facilitate early detection, screening, and referrals to cancer centers; central tertiary oncology referral centers of excellence. Enhancing national cancer registry reporting and promoting cancer research with primary focus on UAE population for cancer epidemiology are other key requirements to achieve advancements in cancer care in the UAE [19, 20].

Despite the substantial health care burden associated with BC, there is paucity of evidence on the disease burden and health care costs associated with the condition in the UAE. The current study aimed to describe the disease burden, health care resource utilization (HCRU), and associated costs among patients diagnosed with BC. The study also evaluated the cost burden due to adverse effects called events of special interest in a subset of the population treated with CDK4/6 inhibitors and studied their cost relative to similar events among patients with BC.

## Methods

### Study design

**Fig. 1:**
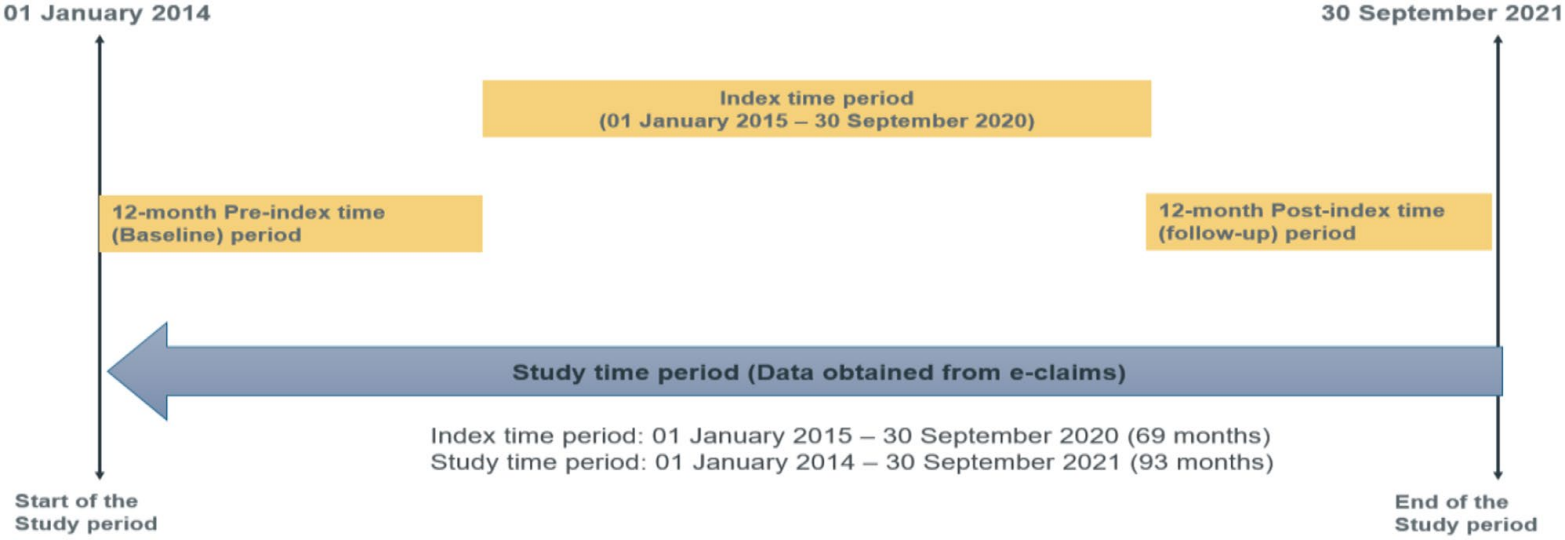
Study design

This was a retrospective analysis of patients diagnosed with BC from the Dubai Real-World Claims Database (DRWD), an e-claims database. The data analyses covered the study period between 01 January 2014 and 30 September 2021. The index date for each patient was defined as the date on which the first diagnosis of BC was identified during the patient identification period or the index period (between 01 January 2015 and 30 September 2020) within the database. The pre-index period was defined as the 12 months prior to the index date and the post-index period as the 12 months following the index date (Fig. 1). The analysis was performed using Structured Query Language (SQL) Server 2014 using the latest version of statistical analysis software (SAS) V9.4.

### Data source

The DRWD e-claims database is the largest claims database of private insurers in the Emirates of Dubai and contains information pertaining to patients’ demographics, diagnoses, procedures (medical, surgical, and diagnostic), prescriptions, and other related services. This database captures approximately 100% of the population covered by Dubai private health insurance. Approximately 80% of the population in Dubai is covered by private insurance, while the remaining 20% is covered by public funding. The Dubai e-claims database represents the multiethnic expat population in Dubai. All local, legal, and regulatory guidelines were followed, with no need for institutional review board (IRB) approval or signing informed consent forms, as these were anonymized patient-level data.

### Study population

The International Classification of Diseases, 10th Revision, Clinical Modification (ICD-10-CM) codes were used to identify patients with diagnoses of advanced/metastatic breast cancer in the database (ICD-10 codes for diagnosis of BC are specified in the appendix in supplementary material). Female patients aged ≥ 18 years (latest available in DRWD at the time of extraction of data) with a BC diagnosis, at least 1 diagnosis claim for any time, and continuous enrollment during the study period were included in this study. Incidental patients were excluded using continuous enrollment as a surrogate, i.e., patients were required to be continuously eligible with at least one claim for any service for 1 year during the pre-index period or 1-year following the index period. Patients with at least one pediatric claim and all male patients were excluded from the study.

For selecting patients with events of special interest, the date of the first diagnosis of BC was considered the index date. From the index date, patients with a diagnosis of any event of special interest throughout the patient journey were identified based on ICD-10 codes and included in the analysis. The special events included cardiac rhythm abnormalities, hepatic events, diarrhea, neutropenia, venous thromboembolism, heart failure, interstitial lung disease, and ischemia and infarction.

Bias: The study population were selected from the DRWD, which comprised predominantly the privately insured expatriate community, representative of the multi-ethnic expat population of Dubai. This limits generalizability to the total population. Also, only direct healthcare costs were evaluated. However, in the study, we have carefully selected patient population and cost comparisons, and tried to overcome bias, and even though there could be some variability in cost, it would not impact the study results adversely.

### Ethical considerations

The study was observational, involving the collection of anonymized structured data previously collected and did not impose any form of intervention. Hence, obtaining informed consent was not a requirement for the study. IRB approval to conduct this study was not required, since it did not involve the collection, use, or transmission of individually identifiable data. The study adhered to the Health Insurance Portability and Accountability Act of 1996 to prevent disclosure of patients’ health information.

### Baseline variables and outcomes

- Demographic and patient data were extracted from pre-index claims records, and the latest available age at the time of data extraction was considered for the analysis. Assessment for the presence of any pre-existing comorbidities was done through the identification of clinically relevant disease conditions using ICD-10 CM codes. Comorbidities were quantified using Quan-Charlson Comorbidity Index [21] scores.
- All-cause and BC-specific HCRU and associated costs were evaluated among BC-diagnosed patients during the post-index period. For all-cause costs, costs incurred for all claims that patient encountered during the 1-year follow-up period were considered for assessment. For BC-specific costs, costs incurred for claims that were specific to BC during the 1-year follow-up period from the index diagnosis were considered for assessment.
- Events of special interest specific to CDK4/6 inhibitors were evaluated during the post-index period among patients with BC and in the subset of the study population treated with CDK4/6 inhibitors. The data for events of special interest were extracted considering the entire patient journey from the index date (date of first diagnosis of BC). The events of special interest considered for evaluation included cardiac rhythm abnormalities, hepatic events, diarrhea, neutropenia, venous thromboembolism, heart failure, interstitial lung disease, and ischemia and infarction.
- The treatment pattern in the subset of patients treated with CDK4/6 inhibitors was evaluated during the post-index period.
- The HCRU and associated costs were also assessed in patients with BC with events and special interest, as well as in the subset of BC patients who experienced events of special interest and treated with CDK4/6 inhibitors. The HCRU and associated costs were determined based on visit type (inpatient, outpatient, and emergency room) and activity type (medications, procedures [medical, surgical, and diagnostic services], consumables [heterogeneous objects, including syringes, needles, gloves, mask etc.], services [administrative and consultation services], and diagnosis-related group [DRG]).

### Statistical analysis

Descriptive statistics were used to analyze the study variables, including demographic characteristics, events of special interest, health care utilization, and cost. The following were used to calculate the HCRU and cost: mean, median, and standard deviation. Age and events of special interest were calculated by frequency and percentage.

## Results

Of the 12,418 ‘potentially eligible’ patients identified from the database, 8,031 met the inclusion criteria and were included in the study for analyses.

### Baseline characteristics of patients diagnosed with breast cancer

Of the 8,031 (100%) patients included during the index period, data for age were available for only one-fourth (*n* = 1,960 [as of 30 September 2021]), with the majority (68.1%) belonging to 41–60-year age group. Eligible patients had a median age (latest available age at data extraction) of 49.0 years (minimum: maximum: 18.0–91.0). Data for nationality were available for 1,945 patients (24.0%). Details of key demographic characteristics of the study population are summarized in Table 1.

**Table 1:** Demographics characteristics of study population at baseline

Baseline characteristics	Patients	
	*N*	%
**Overall study population**	**8031**	**100.0**
Overall study population with age available^a^	1960	24.4
Overall study population with age missing^a^	6071	75.6
**Age (latest available age, in years)—number of patients** ^**b**^		
18–30	33	1.7
31–40	307	15.7
41–50	754	38.5
51–60	581	29.6
61–70	209	10.7
> 70	76	3.9
**Age statistics**		
Mean (SD)	50.0 (10.0)	
Median	49.0	
Minimum	18.0	
Maximum	91.0	
**Nationality**	**8031**	**100**
**Available patient nationality (≥ 1%)** ^**b**^	**1945**	**24.0**
India	424	21.8
Philippines	363	18.7
Britain	153	7.9
Egypt	139	7.1
Pakistan	101	5.2
Syria	72	3.7
Lebanon	72	3.7
Jordan	69	3.5
Emirates	47	2.4
USA	34	1.7
Canada	31	1.6
Iraq	31	1.6
Iran	27	1.4
South Africa	22	1.1
France	22	1.1
Australia	19	1.0
**Baseline risk scores (pre-index period)**		
**Overall study population in pre-index period**	4925	61
**Quan-Charlson comorbidity index**[21]^**c**^		
**Baseline risk scores**		
0	3456	70.2
1–2	1240	25.2
3–4	104	2.1
5–6	53	1.1
7+	72	1.5
**Baseline risk statistics**		
N	4925	
Mean (SD)	1.9 (1.8)	
Median (minimum: maximum)	1.0 (1.0–12.0)	

*CCI* Charlson comorbidity index, *SD* Standard deviation, *USA* United States of America

^a^ Percentages were calculated using the overall study population during index period (*n* = 8,031) as denominator

^b^ Percentages were calculated using the overall study population during index period with age available (*n* = 1,960) as denominator

^c^ Percentages were calculated using the overall study population during pre-index period (*n* = 4,925) as denominator

### Health care resource utilization and costs among patients diagnosed with BC: Overall vs. disease specific (12-month post-index period)

HCRU claims and associated cost data were available for 7,664 (95.4%) patients of the total study population of 8,031 during the post-index period. During the 12-month post-index period, the mean overall all-cause cost was USD 21,704.3 and the mean overall disease-specific cost, USD 18,250.

#### By visit type: all-cause and disease-specific HCRU and associated costs (gross cost)

All-cause and BC-specific mean gross costs for inpatient visits were higher compared with the costs incurred due to outpatient visits and emergency visits (Table 2 and Table S1).

**Table 2:** Disease-specific HCRU and costs (gross cost) by visit type for patients with BC

	Overall (*N*)	Overall (%)
**Overall study population**	8031	100.0
**Number of patients at 1-year follow-up**	7664	95.4
**Health care utilization: Number of visits (claims)**		
**Inpatient**		
N (patient counts)	1835	23.9
Total	3452	
Mean (SD)	1.9 (2.1)	
**Emergency room**		
N (patient counts)	436	5.7
Total	771	
Mean (SD)	1.8 (1.7)	
**Outpatient**		
N (patient counts)	7425	96.9
Total	108,900	
Mean (SD)	14.7 (20.7)	
**Health care cost (in USD)**		
**Inpatient cost**		
N (patient counts)	1835	23.9
Total cost (USD)	31114445.3	
Mean (SD)	16,956.2 (19864.9)	
**Emergency cost**		
N (patient counts)	436	5.7
Total cost (USD)	290283.2	
Mean (SD)	665.8 (1212.3)	
**Outpatient cost**		
N (patient counts)	7425	96.9
Total cost (USD)	108463215.1	
Mean (SD)	146,079 (30391.3)	

Source for conversion of AED to USD currency: https://www.unitconverters.net/currency/aed-to-usd.htm; Accessed on 19 April 2023 10:30:00

1 AED (United Arab Emirates dirham) = 0.2723014922 United States dollar (currency values in USD rounded off to one decimal point)

*HCRU* Health care resource utilization, *SD* Standard deviation, *USD* United States dollar

Percentages were calculated using the number of patients at 1-year follow-up (*n* = 7,664) as denominator

**Visit type**

**Inpatient** (inpatient bed + no emergency room, inpatient bed + emergency room)

**Emergency room visits** (no bed + emergency room, day case bed + emergency room)

**Outpatient** (no bed + no emergency room, day case bed + no emergency room, nationals’ screening, new visa screening, renewal visa screening, home, assisted living facility, mobile unit, ambulance–land, ambulance–air or water)

#### By activity type: all-cause and disease-specific HCRU and associated costs (net cost)

The all-cause net cost was highest for procedures (USD 9,765.5) and lowest consumables (USD 1,553.8) (Table S2). However, the disease-specific net cost was highest for medications (USD 10,251.3) and lowest for consumables (USD 1,502.3), among other activities (Table 3).

**Table 3:** Disease-specific HCRU and costs (net cost) by activity type among patients with BC

	Overall (*N*)	Overall (%)
**Overall study population**	8031	100.0
**Number of patients at 1-year follow-up**	7664	95.4
**Health care utilization: Number of visits (claims)**		
**Medications**		
N (patient counts)	5119	66.8
Total	34,650	
Mean (SD)	6.8 (7.9)	
**CPT procedures**		
N (patient counts)	6767	88.3
Total	74,204	
Mean (SD)	11.0 (16.6)	
**HCPCS (consumables)**		
N (patient counts)	2314	30.2
Total	7824	
Mean (SD)	3.4 (4.7)	
**Services**		
N (patient counts)	6666	87
Total	46,932	
Mean (SD)	7.0 (9.2)	
**DRG**		
N (patient counts)	172	2.2
Total	217	
Mean (SD)	1.3 (0.6)	
**Health care cost (in USD)**		
**Medications cost**		
N (patient counts)	5119	66.8
Total cost (USD)	52476372.4	
Mean (SD)	10251.3 (23274.7)	
**CPT procedures cost**		
N (patient counts)	6,767	88.3
Total cost (USD)	59,713,465	
Mean (SD)	8824.2 (15772.0)	
**HCPCS (consumables) cost**		
N (patient counts)	2314	30.2
Total cost (USD)	3476223.2	
Mean (SD)	1502.3 (3039.7)	
**Services cost**		
N (patient counts)	6666	87.0
Total cost (USD)	12089661.5	
Mean (SD)	1813.5 (3822.6)	
**DRG cost**		
N (patient counts)	172	2.2
Total cost (USD)	1,575183.5	
Mean (SD)	9158.0 (10,457.2)	

Source for conversion of AED to USD currency: https://www.unitconverters.net/currency/aed-to-usd.htm; Accessed on 19 April 2023 10:30:00

1 AED (United Arab Emirates Dirham) = 0.2723014922 United States dollar (currency values in USD rounded off to one decimal point)

*CPT* Current procedural terminology, *DRG* Diagnosis-related group, *HCPCS* Healthcare Common Procedure Coding System, *HCRU* Health care resource utilization, *SD* Standard deviation, *USD* United States dollar

Percentages were calculated using the number of patients at 1-year follow-up (*n* = 7,664) as denominator

During the post-index period, BC-specific costs contributed to 84% (overall mean disease-specific cost/overall mean all-cause cost x 100 [USD 18,250/USD 21,704 × 100]) of the overall disease burden among patients with BC. Two independent factors, viz. inpatient cost (based on visit type) (USD 16,956.2) and medication cost (based on activity type) (USD 10,251.3) during the 12-month follow-up period, contributed significantly to BC-specific costs.

### Health care cost due to events of special interest among patients with BC

During the 12-month post-index period, HCRU costs were available for 2,183 (28.0%) patients with BC with events of special interest. The most commonly (~ 90.0%) recorded events of special interest included cardiac rhythm abnormalities (27.0%), hepatic events (26.0%), diarrhea (20.0%), neutropenia (15.0%), venous thromboembolism (7.0%), and heart failure (4.0%) (Table S3).

#### By visit type: all-cause and disease-specific HCRU and associated costs (gross cost) among patients with BC with events of special interest

**Fig. 2:**
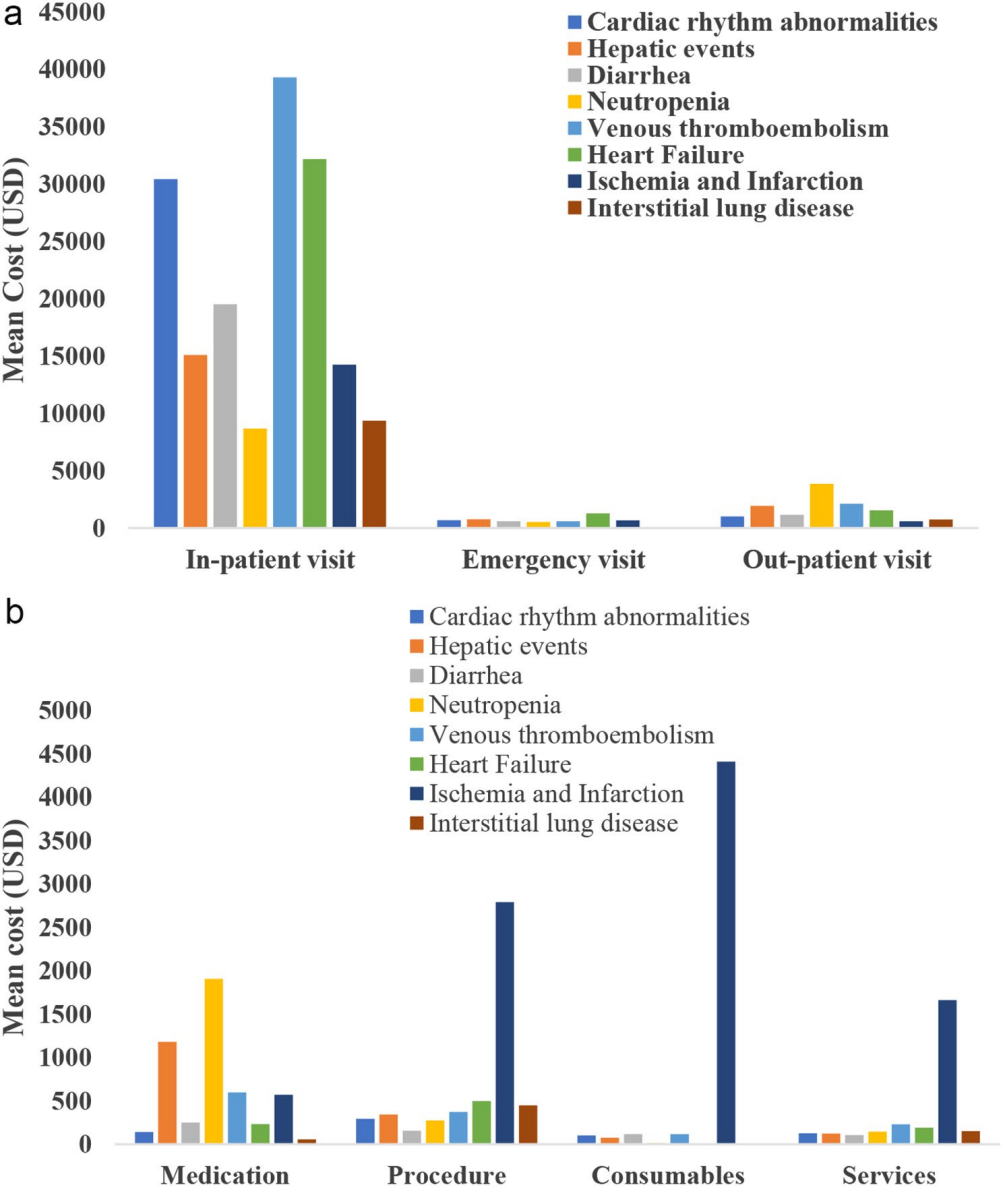
**a**. Disease Specific HCRU and Gross Cost for Event of Interest by Encounter type. **b**. Disease-specific HCRU and Net Cost for Event of Interest by Activity type

All-cause gross costs incurred for outpatient and inpatient visits were higher compared with those for emergency visits for all events of special interest. Both all-cause and BC-specific mean costs incurred for inpatient visit were highest for venous thromboembolism (USD 41,280.6 and USD 39,292.3) (Table 4 and Table S4). The disease-specific mean outpatient cost was highest for neutropenia (USD 3,865.3) (Table 4; Fig. 2a).

**Table 4:** Disease-specific HCRU and costs (gross cost) by visit type for events of special interest

Health care utilization: Number of visits (claims)								
Event of interest	Cardiac rhythm abnormalities	Hepatic events	Diarrhea	Neutropenia	Venous thromboembolism	Heart failure	Ischemia and infarction	Interstitial lung disease
Overall claims	1720	1808	1208	1326	683	441	45	87
**Inpatient visits**								
N (patient counts)	124	62	89	131	38	37	8	3
Total	151	70	110	199	74	62	10	3
Mean (SD)	1.2 (0.5)	1.1 (0.5)	1.2 (0.6)	1.5 (1.7)	1.9 (3.3)	1.7 (1.2)	1.3 (0.5)	1.0
**Emergency room visits**								
N (patient counts)	63	21	84	25	14	6	3	1
Total	87	25	116	29	18	9	3	1
Mean (SD)	1.4 (0.9)	1.2 (0.4)	1.4 (0.8)	1.2 (0.4)	1.3 (0.5)	1.5 (0.5)	1.0	1.0 (-)
**Outpatient visits**								
N (patient counts)	548	595	394	292	145	82	11	26
Total	1482	1713	982	1098	591	370	32	83
Mean (SD)	2.7 (2.9)	2.9 (2.6)	2.5 (2.2)	3.8 (5.3)	4.1 (5.5)	4.5 (4.8)	2.9 (3.2)	3.2 (2.9)
**Healthcare cost (in USD)**								
**Inpatient cost**								
N (patient counts)	124	62	89	131	38	37	8	3
Total cost (USD)	3,771,954	935308.8	1737076.6	1135173.7	1493108.3	1190331.7	113975.3	28080.3
Mean (SD)	30419.1 (42221.4)	15085.5 (25112.7)	19517.7 (27501.6)	8665.4 (10238.8)	39292.3 (60250.5)	32171.1 (45132.1)	14246.8 (14086.2)	9360.1 (7881.8)
**Emergency cost**								
N (patient counts)	63	21	84	25	14	6	3	1
Total cost (USD)	43,284	16193.5	50136.4	13481.4	8330.8	7727.9	1995.4	
Mean (SD)	687.0 (560.9)	771.2 (459.9)	596.9 (504.6)	539.2 (488.0)	595.0 (349.1)	1,288.0 (976.2)	665.2 (703.3)	
**Outpatient cost**								
N (patient counts)	548	595	394	292	145	82	11	26
Total cost (USD)	560117.6	1149163.2	456665.7	1128692.7	309148.8	126259.4	6461.4	19436.3
Mean (SD)	1022.2 (2771)	1931.4 (5036)	1159.0 (2972.2)	3865.3 (10574.6)	2132.1 (4223.3)	1539.9 (1914.3)	587.3 (1109.9)	747.5 (694.1)

Source for conversion of AED to USD currency: https://www.unitconverters.net/currency/aed-to-usd.htm; Accessed on 19 April 2023 10:30:00

1 AED (United Arab Emirates dirham) = 0.2723014922 United States dollar (currency values in USD rounded off to one decimal point)

*CPT* Current procedural terminology, *DRG* Diagnosis-related group, *HCPCS* Healthcare Common Procedure Coding System, *HCRU* Health care resource utilization, *SD* Standard deviation, *USD* United States dollar

#### All-cause and disease-specific health care resource utilization and associated costs among patients with BC with events of special interest by activity type

In patients with BC and associated events of special interest, mean all-cause claims for medications, procedures, and services were higher compared with those for consumables and DRG (Table S5). The BC-specific net cost for medications was higher among patients with neutropenia (USD 1,906.1). The mean cost due to services and consumables was higher among patients with ischemia and infarction (USD 1,661.6, USD 4,411.3, respectively), while the mean cost for DRG was higher among patients with venous thromboembolism (USD 61,788.2) (Table S6 and Fig. 2b).

During the post-index period among patients with BC, health care costs due to events of special interest (*n* = 1,843) contributed to 17% (mean event of interest disease-specific cost/mean event of interest all-cause cost x 100 [USD 7,760.3/USD 46.198.0 × 100]) of the overall disease burden.

### Treatment pattern and health care costs due to events of special interest in subset of population treated with CDK4/6 inhibitors

#### Treatment pattern

In the subgroup of patients in whom CDK4/6 inhibitors were a part of the regimen (*n* = 174), CDK4/6 inhibitors were commonly prescribed in combination with aromatase inhibitors (41.4%) and estrogen receptor antagonists (17.9%). CDK4/6 inhibitors were also prescribed as monotherapy to 17.1% of patients.

#### HCRU and costs due to events of special interest

Data for events of special interest among patients treated with CDK4/6 inhibitors were available for only 73 patients (77.7%) of the overall study population (*n* = 7,698) during the post-index period. The percentages of events of special interest observed in study population treated with CDK4/6 inhibitors during the post-index period were as follows: neutropenia (30.2%), diarrhea (22.4%), hepatic events (20.7%), cardiac rhythm abnormalities (19.8%), venous thromboembolism (4.3%), heart failure (0.9%), and interstitial lung disease (1.7%) (Table S7).

Mean BC-specific outpatient claims and costs were highest for patients with neutropenia (mean claims = 11.2, mean HCRU cost = USD 20,027.5) (Table 5). High costs were incurred for inpatient visits for patients presenting with cardiac rhythm abnormalities (USD 42,375), diarrhea (USD 20,265.2), and hepatic events (USD 13,987.3).

**Table 5:** Disease-specific HCRU and CDK 4/6 cost by visit type for events of special interest

Health care utilization: number of visits (claims)							
Event of interest	Cardiac rhythm abnormalities	Hepatic events	Diarrhea	Neutropenia	Venous thromboembolism	Heart failure	Interstitial lung disease
**Overall claims**	49	106	104	356	27	5	8
**Inpatient visits**							
N (Patient counts)	10	5	8	8		1	
Total	13	6	9	10		5	
Mean (SD)	1.3 (0.5)	1.2 (0.4)	1.1 (0.4)	1.3 (0.7)		5.0 (-)	
**Emergency room visits**							
N (Patient counts)	2	1	4		1		
Total	2	1	4		1		
Mean (SD)	1.0 (0.0)		1.0 (0.0)				
**Outpatient visits**							
N (Patient counts)	14	21	23	31	5		2
Total	34	99	91	346	26		8
Mean (SD)	2.4 (1.5)	4.7 (4.6)	4.0 (4.5)	11.2 (11.3)	5.2 (6.1)		4.0 (4.2)
**Health care cost (in USD) (gross cost)**							
**Event of interest**	**Cardiac rhythm abnormalities**	**Hepatic events**	**Diarrhea**	**Neutropenia**	**Venous thromboembolism**	**Heart failure**	**Interstitial lung disease**
**Inpatients cost**							
N (patient counts)	10	5	8	8	-		-
Total cost (USD)	423749.9	69936.8	162121.5	102416.1	-		-
Mean (SD)	42375.0 (43456.3)	13987.3 (10695.5)	20265.2 (36126.8)	12802.0 (10549.5)	-		-
**Emergency cost**							
N (patient counts)	2	1	4	-	1	-	-
Total cost (USD)	789.1	401.1	1,427.1	-	30	-	-
Mean (SD)	394.6 (308.5)	401.1 (-)-	356.7 (83.9)	-	29.9 (-)	-	-
**Outpatient cost**							
N (patient counts)	14	21	23	31	5	-	2
Total cost (USD)	19127.5	349340.2	176813.3	620852.8	11260.5	-	1353.6
Mean (SD)	1366.1 (2023.2)	16635.2 (18224.9)	7687.6 (11323.4)	20027.5 (22486.1)	2252.2 (3897.7)	-	676.7 (921.7)

Source for conversion of AED to USD currency: https://www.unitconverters.net/currency/aed-to-usd.htm; Accessed on 19 April 2023 10:30:00

1 AED (United Arab Emirates dirham) = 0.2723014922 United States dollar (currency values in USD rounded off to one decimal point)

*CPT* Current procedural terminology, *DRG* Diagnosis-related group, *HCPCS* Healthcare Common Procedure Coding System, *HCRU* Health care resource utilization, *SD* Standard deviation, *USD* United States dollar

The mean number of BC-specific outpatient claims was higher for neutropenia, while the mean number of inpatient claims were higher for patients with heart failure. Costs incurred for BC-specific outpatient visits were highest among patients with neutropenia (USD 20,027.5), while BC-specific inpatient visits were highest among patients with cardiac rhythm abnormalities (USD 42,375.0) (Table 5).

The mean BC-specific HCRU cost due to medications was highest among patients with hepatic events (USD 14,601.9) and neutropenia (USD 12,837.4). The mean BC-specific HCRU cost due to CPT (procedures) was highest among patients with neutropenia (USD 890.4), followed by hepatic events (USD 670.6) and venous thromboembolism (USD 669.3) (Table 6).

**Table 6:** Disease-specific HCRU and CDK 4/6 cost by activity type for events of special interest

Health care utilization: number of visits (claims)							
Event of interest	Cardiac rhythm abnormalities	Hepatic events	Diarrhea	Neutropenia	Venous thromboembolism	Heart failure	Interstitial lung disease
**Overall claims**	49	106	104	356	27	5	8
**Medications**							
N (patient counts)	14	14	22	28	3	1	2
Total	17	35	43	159	9	1	4
Mean (SD)	1.2 (0.4)	2.5 (2.2)	2.0 (1.6)	5.7 (5.5)	3.0 (2.6)	1.0 (-)	2.0 (1.4)
**CPT procedures**							
N (patient counts)	10	18	19	30	4	1	1
Total	18	48	44	132	9	3	1
Mean (SD)	1.8 (0.9)	2.7 (1.6)	2.3 (2.3)	4.4 (4.2)	2.3 (1.3)	3.0 (-)	1.0 (-)
**HCPCS (consumables)**							
N (patient counts)		2	2	1		1	-
Total		2	2	1		1	-
Mean (SD)		1.0 (0.0)	1.0 (0.0)	1.0 (-)		1.0 (-)	-
**Services**							
N (patient counts)	10	9	8	21	3		1
Total	14	21	14	63	9		3
Mean (SD)	1.4 (0.7)	2.3 (1.7)	1.8 (1.8)	3.0 (2.6)	3.0 (2.6)		3.0 (-)
**DRG**							
N (patient counts)	-	-	1	1	-	-	-
Total	-	-	1	1	-	-	-
Mean (SD)	-	-	1.0 (-)	1.0 (-)	-	-	-
**Health care cost (in USD)**							
**Event of interest**	**Cardiac rhythm abnormalities**	**Hepatic events**	**Diarrhea**	**Neutropenia**	**Venous thromboembolism**	**Heart failure**	**Interstitial lung disease**
**Medications cost**							
N (patient counts)	14	14	22	28	3	1	2
Total cost	7869.8	204426.5	76230.3	359445.6	2430.6		25.6
Mean (SD)	562.0 (1830.7)	14601.9 (13249.6)	3465 (5804.4)	12837.4 (14464.4)	810.1 (1338.4)		12.8 (13.1)
**CPT procedure cost**							
N (patient counts)	10	18	19	30	4	1	1
Total cost	5257.3	12,073	9501.4	26709.2	2677.3		
Mean (SD)	525.8 (814.0)	670.6 (1131.1)	500.0 (591.4)	890.4 (1619.6)	669.3 (858.3)		
**HCPCS (consumables) cost**							
N (patient counts)	-	2	2	1	-	1	-
Total	-	21.2	7.3		-		-
Mean (SD)	-	10.6 (9.3)	3.5 (1.1)		-		-
**Services cost**							
N (patient counts)	10	9	8	21	3	-	1
Total cost	1173.1	2081.5	1250.7	6127.1	728.1	-	
Mean (SD)	117.4 (100.2)	231.2 (158.2)	156.3 (125.3)	291.6 (267.7)	242.7 (203.4)	-	
**DRG cost**							
N (patient counts)	-	-	1	1	-	-	-
Total	-	-			-	-	-
Mean (SD)	-	-			-	-	-

Source for conversion of AED to USD currency: https://www.unitconverters.net/currency/aed-to-usd.htm; Accessed on 19 April 2023 10:30:00

1 AED (United Arab Emirates dirham) = 0.2723014922 United States dollar (currency values in USD rounded off to one decimal point)

*CPT* Current procedural terminology, *DRG* Diagnosis-related group, *HCPCS* Healthcare Common Procedure Coding System, *SD* Standard deviation, *USD* United States dollar

*Disease-specific health care costs due to events of special interest: Overall BC patients versus subset of patients with BC treated with CDK4/6 inhibitors*.

Disease-specific costs due to events of special interest among patients with BC contributed to 43% of the BC cost burden. Disease-specific costs for events of special interest in the subset of patients treated with CDK4/6 inhibitors contributed to 69% of the BC cost burden.

*Disease-specific health care costs due to events of special interest: Overall BC patients versus subset of patients with BC treated with CDK4/6 inhibitors*.

Disease-specific costs due to events of special interest among patients with BC contributed to 43% of the BC cost burden. Disease-specific costs for events of special interest in the subset of patients treated with CDK4/6 inhibitors contributed to 69% of the BC cost burden.

## Discussion

In current study, most of the patients belonged to the age group of 40–60 years, with a median age of 49 years. The findings are in alignment with studies highlighting that the female population in the UAE develop BC a decade earlier compared with their counterparts in the UK and other western countries (median age at BC diagnosis is ~ 60–65 years) [22]. In a population-based case-control study, the median age of patients at clinical diagnosis in the UK was 59.8 years [23]. However, in the US, the median age of patients was 61 years, as determined in a retrospectively analysis using the National Cancer Database [24].

In our study, we analyzed treatment patterns only in subset of patients with BC treated with CDK4/6 inhibitors and found that CDK4/6 inhibitors were more commonly prescribed in combination with aromatase inhibitors and estrogen inhibitors, echoing findings from recent studies. In a retrospective observational study conducted using a Japanese claims database, CDK4/6 inhibitors were found to be prescribed most commonly in combination with estrogen inhibitors (59%) in the context of HR+, HER2-negative advanced BC [25]. A retrospective chart review of patients with HR+, HER2-negative metastatic BC conducted at a cancer center in Washington reported that the CDK4/6 inhibitor palbociclib was prescribed most frequently in combination of with the aromatase inhibitor letrozole (73.5%) [26].

In our study, inpatient costs and medication costs contributed to an increased economic burden, probably due to the younger study population. In a previous retrospective analysis, the implementation rate of breast-conserving surgery and breast reconstruction surgery was found to be higher among young patients (< 40 years) diagnosed with BC versus older patients [27]. Previous literature suggests that younger women (< 45 years of age) have a higher proportion of advanced-stage cancers and may require more intensive treatment. A retrospective study reported that prescription drug costs were higher among patients with BC aged < 45 years compared with older women (USD 2,544 vs. USD 1,771). Excess costs for all services (inpatient, outpatient, prescription drugs) for younger and older women at 12 months were USD 97,486 (95% confidence interval [CI] USD 93,631–101,341) and USD 75,737 (95% CI USD 73,962–77,512), respectively [28]. In a retrospective analysis of patients with advanced BC, the direct cost for advanced BC treatment was observed to be in the range of €29,803 to €92,272, largely attributable to systemic therapies and inpatient visits [29]. In Medicare Claims database study, conducted in the US, the 10-year cost of cancer treatment ranged from $103,573 for stage 0 cancers to $376,573 for stage 4 cancers [30].

In our study, we evaluated the cost of events of special interest caused due to medications for BC. To the best of our knowledge, our study is one of the first studies to evaluate the cost of events of special interest among patients diagnosed with BC using a claims database source in Dubai, UAE. We considered CDK4/6 inhibitor as the index drug and evaluated the cost burden due to events of special interests among patients with BC and in the subset of the study population treated with CDK4/6 inhibitors. Events of special interest we evaluated were specific to CDK4/6 inhibitor therapy and included cardiac rhythm abnormalities, hepatic events, neutropenia, venous thromboembolism, etc. The findings revealed that costs due to events of special interest among patients with BC contributed to 43% of the BC-specific cost burden. Events of interest in the subset of the population treated only with CDK4/6 inhibitors contributed to 69% of the BC-specific cost burden.

While claims data are extremely valuable for the efficient and effective examination of health care outcomes, treatment patterns, resource utilization, and costs, they are collected for the purposes of payment and not research. Therefore, certain limitations are associated with claims database use. In our study, the study sample covered only the privately insured population in the UAE (Dubai). This limited the generalizability of the data to the entire population. Since the majority of the population in this database comprised expats, there is a possibility that the first diagnosis may not have been captured.Also, treatment data before the index date may not be available, thus historical data on the line of therapies or procedures prior to the index date could not be analyzed. The DRWD data does not support direct one to one mapping between diagnosis and medication/procedure/HCPCS (supplies and consumables) /services, hence the reported cost may or may not be related directly to the BC. Only direct healthcare costs were evaluated; indirect costs, such as work productivity and quality of life, were not included in the evaluation of the economic burden of patients with BC. However, in the study, we have tried to capture the most indicative cost despite these limitations, by careful patient selection and cost comparisons where applicable.

Our study was one of the first to report the cost burden of events of special interest among patients with BC, and the findings suggest that costs due to events of special interest are an additional burden on health care costs among patients with BC. In this study, we attempted to provide the healthcare cost in patients with BC in the overall population and the cost of side effect profile of a representative commonly used drug to treat BC with a distinctive adverse event profile. This would help readers compare these costs for the first time and facilitate informed choices of cost of treatment. It would help them discuss this cost with patients in the treatment decisions and help to better understand cost of treatment care in these settings. Payers should support and implement future policy measures aimed at reducing the cost burden in BC patients.

The study was conducted using the DRWD, which represents the private health care sector in the Emirate of Dubai, constituting approximately 2.3 million individuals and accounting for approximately 28.0% of the UAE population [31]. Therefore, the findings of the current study are expected to address prevalent knowledge gaps pertaining to the health care burden and cost of treatment of BC in the UAE region. among patients with BC.

## Conclusion

BC is one of the most frequently occurring cancers among women with a substantial disease burden in the UAE. The current study findings showed that 55.9% of patients diagnosed with BC were less than 50 years of age. Therefore, risk assessment for BC should be recommended in clinics to determine early-stage BC and, accordingly, health care policy makers should implement suitable measures. The study reports a significant cost burden among patients with BC, with BC-specific costs contributing to 84% of the overall disease cost burden. Inpatient and medication costs were major contributors to BC-specific costs. The study highlights that events of special interest contribute to 43% of the disease-specific cost burden. These study findings can inform key health care stakeholders (payers and providers) on future policy measures aiming to reduce the cost burden due to hospitalization, medications, and costs due to events of special interest among patients with BC. Hence, future research on the long-term safety and efficacy of novel targeted therapies will help payers effectively use available resources and reduce the economic burden. The UAE has its own consensus on BC, which was held in September 2023 and arranged by the Emirates Oncology Society. The findings will be published soon, as per the regional and demographic data specific to the UAE.

### Electronic supplementary material

Below is the link to the electronic supplementary material.

Supplementary Material 1

## Data Availability

The data used to support the findings of this study are included within the manuscript file and the supplementary table file.

## Abbreviations

AED: United Arab Emirates dirham
ADP: Adenosine Diphosphate
BC: Breast Cancer
CCI: Charlson Comorbidity Index
CDK: Cyclin-Dependent Kinase
CPT: Current Procedural Terminology
DRG: Diagnosis Related Group
DRWD: Dubai Real-World Database
ER: Estrogen Receptor
HCPCS: Healthcare Common Procedure Coding System
HER2: Human epidermal growth factor Receptor 2
HCRU: Health Care Resource Utilization
ICD-10-CM: International Classification Of Diseases, 10th Revision, Clinical Modification
ICF: Informed Consent Form
IRB: Institutional Review Board
mTOR: Mammalian Target Of Rapamycin
PARP: Poly Adenosine Diphosphate-Ribose
PR: Progesterone Receptor
SAS: Statistical Analysis Software
SQL: Structured Query Language
USD: United States dollar
