# Supplementary material for: A retrospective cohort study to evaluate disease burden, health care resource utilization, and costs in patients with breast cancer in Dubai, UAE
Source: BMC Health Serv Res. 2024 Jul 12;24:810. doi: 10.1186/s12913-024-11193-8 (PMC11241818; doi:10.1186/s12913-024-11193-8)
Supplement: Supplementary file 1 — Supplementary Material 1 [file 12913_2024_11193_MOESM1_ESM.docx]

**Table S1** All-cause healthcare resource utilization and costs (gross cost) by visit type for breast cancer patients

|  | **Overall (N)** | **Overall (%)** |
| --- | --- | --- |
| **Overall study population** | 8031 | 100.0 |
| **Number of patients in 1-year follow up** | 7698 | 96 |
| **Healthcare utilization: number of visits (claims)** | | |
| **In-patient** |  |  |
| N (patient counts) | 2304 | 30 |
| Total | 4481 |  |
| Mean (SD) | 1.9 (2.3) |  |
| **Emergency room** |  |  |
| N (patient counts) | 1429 | 18.6 |
| Total | 3332 |  |
| Mean (SD) | 2.3 (2.3) |  |
| **Out-patient** |  |  |
| N (patient counts) | 7520 | 97.9 |
| Total | 181602 |  |
| Mean (SD) | 24.1 (26.0) |  |
| **Healthcare cost (in USD)** | | |
| **In-patient cost** |  |  |
| N (patient counts) | 2304 | 30 |
| Total | 39279157.2 |  |
| Mean (SD) | 17048.3 (20580.8) |  |
| **Emergency cost** |  |  |
| N (patient counts) | 1429 | 18.6 |
| Total cost (USD) | 1049818.9 |  |
| Mean (SD) | 734.7 (1014.9) |  |
| **Out-patient cost** |  |  |
| N (patient counts) | 7520 | 97.9 |
| Total cost (USD) | 126468322.6 |  |
| Mean (SD) | 16817.6 (31443.7) |  |

Source for conversion of AED to USD currency: https://www.unitconverters.net/currency/aed-to-usd.htm; Accessed on 19 April 2023 10:30:00

1 AED (United Arab Emirates Dirham) = 0.2723014922 United States dollar (currency values in USD rounded off to one decimal point)

Abbreviations: USD-United States dollar; SD-Standard deviation.

Percentages were calculated using the number of patients in 1-year follow- up (n=7,698) as denominator.

**Encounter Type**

**In-patient** (in-patient Bed + no emergency room, in-patient bed+emergency room).

**Emergency room Visits** (no Bed + emergency room, day case bed + emergency room).

**Out-patient** (No bed + no emergency room, day case Bed + no emergency room, nationals screening, new visa screening, renewal visa screening, home, assisted living facility, mobile unit, ambulance - land, ambulance - air or water)

**Note**: Patient can present in more than one encounter, hence the patient counts are not mutually exclusive.

Excluded the patients/ claims having amount zero, due to this we see a slight difference in the patient counts in each cohort.

Gross cost reported is paid by the insurance+patient.

**Table S2** All-cause healthcare resource utilization and costs (net cost) by activity type for breast cancer patients

|  | **Overall (N)** | **Overall (%)** |
| --- | --- | --- |
| **Overall study population** | 8031 | 100.0 |
| **Number of patients in 1-year follow up** | 7685 | 95.7 |
| **Healthcare Utilization: Number of Visits (Claims)** | | |
| **Medications** | | |
| N (patient counts) | 6761 |  |
| Total | 61524 |  |
| Mean (SD) | 9.1 (9.7) |  |
| **CPT procedures** | | |
| N (patient counts) | 7380 |  |
| Total | 108390 |  |
| Mean (SD) | 14.7 (18.4) |  |
| **HCPCS (consumables)** | | |
| N (patient counts) | 3126 |  |
| Total | 10636 |  |
| Mean (SD) | 3.4 (5.1) |  |
| **Services** | | |
| N (patient counts) | 7329 |  |
| Total | 79258 |  |
| Mean (SD) | 10.8 (11.2) |  |
| **DRG** | | |
| N (patient counts) | 238 |  |
| Total | 321 |  |
| Mean (SD) | 1.3 (0.6) |  |
| **Healthcare cost (in USD)** | | |
| **Medications cost** | | |
| N (patient counts) | 6761 |  |
| Total cost (USD) | 57784205.2 |  |
| Mean (SD) | 8546.7 (21360.4) |  |
| **CPT procedures cost** | | |
| N (patient counts) | 7380 |  |
| Total cost (USD) | 72068818.5 |  |
| Mean (SD) | 9765.5 (16298.6) |  |
| **HCPCS (Consumables) cost** | | |
| N (patient counts) | 3126 |  |
| Total cost (USD) | 4857428.7 |  |
| Mean (SD) | 1553.8 (3187.3) |  |
| **Services cost** | | |
| N (patient counts) | 7329 |  |
| Total cost (USD) | 16530772.5 |  |
| Mean (SD) | 2255.5 (4260.2) |  |
| **DRG cost** | | |
| N (patient counts) | 238 |  |
| Total cost (USD) | 2232497.3 |  |
| Mean (SD) | 9380.2 (10281.6) |  |

Source for conversion of AED to USD currency: https://www.unitconverters.net/currency/aed-to-usd.htm; Accessed on 19 April 2023 10:30:00

1 AED (United Arab Emirates Dirham) = 0.2723014922 United States dollar (currency values in USD rounded off to one decimal point)

CPT-Current procedural terminology, DRG-Diagnosis related group, HCPCS-Healthcare common procedure coding system; SD-Standard deviation.

Percentages were calculated using the number of patients in 1-year follow-up (n=7685) as denominator.

**Note**: 1. Patient can present in more than one encounter, hence the patient counts are not mutually exclusive.

2. Excluded the patients/Claims having claim amount zero.

3. Net cost reported is paid by the payer.

**Table S3** Events of special interest among patients with B during post-index period

| **Population** | **Number of Patients** | |
| --- | --- | --- |
| Overall Study Population in Index Period | 8031 (100.0%) | |
| Overall BC Diagnosed patients in 12-month Post-index Period | 7698 (95.8%) | |
| BC diagnosed patients who had event of Special Interest during the 12-month post-index follow‑up period | 2183 (28.0%) | |
| Overall event of interest patients during the 1-year follow-up period meeting continuous enrollment criteria. | 1843 (84.0%) | |
| **Events of Special Interest in the 1 year Follow-up Period** | **Number (%) of patients** | **Number of claims** |
| Cardiac rhythm abnormalities^a^ | 666 (27.0%) | 18810 |
| Hepatic events | 652 (26.0%) | 19899 |
| Diarrhea | 497 (20.0%) | 17696 |
| Neutropenia | 369 (15.0%) | 16375 |
| Venous thromboembolism | 173 (7.0%) | 5926 |
| Heart failure | 105 (4.0%) | 2820 |
| Interstitial lung disease | 29 (1.0%) | 458 |
| Ischemia and infarction | 20 (1.0%) | 1398 |

BC-Breast cancer.

**Note**: Patients are not mutually exclusive.

^a^ Cardiac rhythm abnormalities group included abnormalities of heart rhythm, arrhythmia, atrial fibrillation and flutter, cardiac arrhythmias, and heart block in this study.

**Table S4** All-cause healthcare resource utilization and costs (gross cost) by visit type for specific events of interest

| **Healthcare utilization: number of visits (claims)** | | | | | | | | |
| --- | --- | --- | --- | --- | --- | --- | --- | --- |
| **Event of Interest** | **Cardiac rhythm abnormalities** | **Hepatic events** | **Diarrhea** | **Neutropenia** | **Venous thromboembolism** | **Heart Failure** | **Ischemia and Infarction** | **Interstitial lung disease** |
| **Overall claims** | 18810 | 19899 | 17696 | 16375 | 5926 | 2820 | 458 | 1398 |
| **In-patients visit** | | | | | | | | |
| N (patient counts) | 252 | 220 | 191 | 235 | 70 | 52 | 13 | 10 |
| Total | 561 | 529 | 490 | 610 | 184 | 113 | 47 | 22 |
| Mean (SD) | 2.2 (3.7) | 2.4 (3.4) | 2.6 (3.5) | 2.6 (2.6) | 2.6 (3.7) | 2.2 (1.8) | 3.6 (7.1) | 2.2 (1.4) |
| **Emergency room visits** | | | | | | | | |
| N (patient Counts) | 203 | 164 | 172 | 127 | 49 | 32 | 9 | 10 |
| Total | 524 | 364 | 488 | 360 | 108 | 96 | 15 | 23 |
| Mean (SD) | 2.6 (2.7) | 2.2 (2.3) | 2.8 (3.1) | 2.8 (2.5) | 2.2 (1.6) | 3.0 (3.1) | 1.7 (0.7) | 2.3 (2.1) |
| **Out-patient Visits** | | | | | | | | |
| N (patient counts) | 622 | 643 | 479 | 350 | 164 | 92 | 14 | 28 |
| Total | 17725 | 19006 | 16718 | 15405 | 5634 | 2611 | 396 | 1353 |
| Mean (SD) | 28.5 (27.7) | 29.6 (31.5) | 34.9 (38.4) | 44.0 (38.8) | 34.4 (31.9) | 28.4 (24.9) | 28.3 (22.0) | 48.3 (36.3) |
| **Healthcare cost (In USD)** | | | | | | | | |
| **Event of Interest** | **Cardiac rhythm abnormalities** | **Hepatic events** | **Diarrhea** | **Neutropenia** | **Venous thromboembolism** | **Heart Failure** | **Ischemia and Infarction** | **Interstitial lung disease** |
| **In-patients cost** | | | | | | | | |
| N (patient counts) | 252 | 220 | 191 | 235 | 70 | 52 | 13 | 10 |
| Total cost (USD) | 6832605.9 | 4790000.3 | 4689740.2 | 5013870.5 | 2889638.7 | 1670681.3 | 209349.5 | 234548.0 |
| Mean (SD) | 27113.6  (36508.3) | 21772.7 (29875.3) | 24553.7  (31329.1) | 21335.6  (25463.2) | 41280.6  (54856.0) | 32128.6  (40805.5) | 16103.9 (14030.6) | 23454.7 (31214.5) |
| **Emergency cost** | | | | | | | | |
| N (patient counts) | 203 | 164 | 172 | 127 | 49 | 32 | 9 | 10 |
| Total cost  (USD) | 191929.5 | 121192.4 | 182299.9 | 123871.3 | 39390.6 | 40283.7 | 5743.7 | 8787.2 |
| Mean (SD) | 945.4  (1626.5) | 739.0  (810.9) | 1059.8  (1384.7) | 975.4  (1005.9) | 803.8  (756.5) | 1258.8 (1182.6) | 638.3 (573.7) | 878.7 (875.7) |
| **Out-patient Cost** | | | | | | | | |
| N (patient counts) | 622 | 643 | 479 | 350 | 164 | 92 | 14 | 28 |
| Total cost  (USD) | 10058191.1 | 12176837.2 | 13667820.8 | 14886687.2 | 4070533.2 | 1767763.6 | 139971.7 | 1331210.4 |
| Mean (SD) | 16170.6 (28103.4) | 18937.5  (31893.9) | 28534.2  (47510.3) | 42533.5  (46645.5) | 24820.3  (38456.3) | 19215.0  (27127.5) | 9998.1  (12484.5) | 47543.3  (64578.7) |

Source for conversion of AED to USD currency: https://www.unitconverters.net/currency/aed-to-usd.htm; Accessed on 19 April 2023 10:30:00

1 AED (United Arab Emirates Dirham) = 0.2723014922 United States dollar (currency values in USD rounded off to one decimal point)

USD-United States dollar; SD-Standard deviation.

**Note**: Patient can present in more than one encounter, hence the patient counts are not mutually exclusive

**Table S5** All-Cause Healthcare Resource Utilization and Costs (Net Cost) By Activity Type for Specific Events of Interest

| **Healthcare Utilization: Number of Visits (Claims)** | | | | | | | | |
| --- | --- | --- | --- | --- | --- | --- | --- | --- |
| **Event of Interest** | **Cardiac rhythm abnormalities** | **Hepatic events** | **Diarrhea** | **Neutropenia** | **Venous thromboembolism** | **Heart Failure** | **Ischemia and Infarction** | **Interstitial lung disease** |
| **Overall Claims** | 18810 | 19899 | 17696 | 16375 | 5926 | 2820 | 458 | 1398 |
| **Medications** | | | | | | | | |
| N (patient counts) | 598 | 587 | 459 | 341 | 160 | 92 | 21 | 26 |
| Total | 5338 | 5996 | 5424 | 4291 | 1840 | 834 | 214 | 370 |
| Mean (SD) | 8.9 (10.6) | 10.2 (17.1) | 11.8 (23.8) | 12.6 (11.5) | 11.5 (12.2) | 9.1 (8.2) | 10.2 (11) | 14.2 (10.9) |
| **CPT Procedures** | | | | | | | | |
| N (patient counts) | 604 | 620 | 463 | 354 | 159 | 98 | 20 | 26 |
| Total | 7156 | 7923 | 7047 | 7452 | 2229 | 1013 | 288 | 611 |
| Mean (SD) | 11.8 (15.7) | 12.8 (15.1) | 15.2 (18.1) | 21.1 (21.2) | 14.0 (15.3) | 10.3 (11.4) | 14.4 (18.2) | 23.5 (20.7) |
| **HCPCS (Consumables)** | | | | | | | | |
| N (patient counts) | 134 | 143 | 107 | 107 | 47 | 18 | 12 | 9 |
| Total | 377 | 459 | 374 | 559 | 166 | 45 | 44 | 21 |
| Mean (SD) | 2.8 (5.4) | 3.2 (8.8) | 3.5 (6.2) | 5.2 (13.5) | 3.5 (3.9) | 2.5 (2.2) | 3.7 (4.8) | 2.3 (2.2) |
| **Services** | | | | | | | | |
| N (patient counts) | 601 | 613 | 459 | 338 | 158 | 92 | 20 | 27 |
| Total | 5733 | 5353 | 4732 | 3995 | 1645 | 893 | 202 | 380 |
| Mean (SD) | 9.5 (8.6) | 8.7 (7.6) | 10.3 (9.8) | 11.8 (9.8) | 10.4 (9.5) | 9.7 (9.1) | 10.1 (10.8) | 14.1 (8.6) |
| **DRG** | | | | | | | | |
| N (patient counts) | 19 | 24 | 15 | 14 | 3 | 3 | 1 | 1 |
| Total | 29 | 40 | 20 | 28 | 7 | 9 | 2 | 1 |
| Mean (SD) | 1.5 (0.9) | 1.7 (1.0) | 1.3 (0.8) | 2.0 (1.4) | 2.3 (2.3) | 3.0 (1.0) |  |  |
| **Healthcare Cost (In USD)** | | | | | | | | |
| **Event of Interest** | **Cardiac rhythm abnormalities** | **Hepatic events** | **Diarrhea** | **Neutropenia** | **Venous thromboembolism** | **Heart Failure** | **Ischemia and Infarction** | **Interstitial lung disease** |
| **Medications** | | | | | | | | |
| N (patient counts) | 598 | 587 | 459 | 341 | 160 | 92 | 21 | 26 |
| Total cost (USD) | 1092041.4 | 1826566.0 | 2104894.3 | 2001271.6 | 678458.2 | 150888.8 | 78147.5 | 256643.6 |
| Mean (SD) | 1826.1  (6976.4) | 3111.6  (9658.5) | 4585.8  (14311.1) | 5868.9  (13422.6) | 4240.3  (11247.7) | 1640.1  (2681.6) | 3721.3  (6066.9) | 9870.9 (18084.4) |
| **CPT Procedures** | | | | | | | | |
| N (patient counts) | 604 | 620 | 463 | 354 | 159 | 98 | 20 | 26 |
| Total cost (USD) | 2162894.8 | 2426005.9 | 2474363.9 | 3179491.9 | 710793.5 | 346941.8 | 174065.5 | 291938.2 |
| Mean (SD) | 3581.0  (7405.8) | 3913.0  (8293.5) | 5344.2  (10099.1) | 8981.6  (13109.1) | 4470.4  (8071.0) | 3540.2  (7095.4) | 8703.3  (9035.2) | 11228.4  (14033.3) |
| **HCPCS (Consumables)** | | | | | | | | |
| N (patient counts) | 134 | 143 | 107 | 107 | 47 | 18 | 12 | 9 |
| Total cost (USD) | 38600.9 | 28901.0 | 43303.3 | 52323.5 | 11488.1 | 3831.0 | 39850.0 | 1189.7 |
| Mean (SD) | 288.1  (561.2) | 202.0  (456.4) | 404.6  (1888.4) | 489.1  (2061.0) | 244.5  (406.5) | 212.9  (343.6) | 3,320.7  (5687.8) | 132.1  (169.4) |
| **Services** | | | | | | | | |
| N (patient counts) | 601 | 613 | 459 | 338 | 158 | 92 | 20 | 27 |
| Total cost (USD) | 503173.9 | 482725.2 | 471029.8 | 400849.6 | 151030.1 | 83622.4 | 79058.1 | 41353.3 |
| Mean (SD) | 837.3  (996.4) | 787.5 (905.1) | 1026.3 (1383.0) | 1185.9 (1396.4) | 955.8 (968.8) | 908.9 (980.8) | 3953.0 (4451.3) | 1531.7 (1197.6) |
| **DRG** | | | | | | | | |
| N (patient counts) | 19 | 24 | 15 | 14 | 3 | 3 | 1 | 1 |
| Total cost  (USD) | 260673.7 | 423889.8 | 128501.5 | 295612.7 | 158716.1 | 43495.0 | 29350.6 | 7229.3 |
| Mean (SD) | 13719.6 (23222.4) | 17662.0 (22121.2) | 8566.9 (4284.7) | 21115.1 (36364.0) | 52905.5 (80857.7) | 14498.4 (2187.1) | 29350.6 (-) | 7229.3  (-) |

Source for conversion of AED to USD currency: https://www.unitconverters.net/currency/aed-to-usd.htm; Accessed on 19 April 2023 10:30:00

1 AED (United Arab Emirates Dirham) = 0.2723014922 United States dollar (currency values in USD rounded off to one decimal point)

CPT-Current procedural terminology, DRG-Diagnosis related group, HCPCS-Healthcare common procedure coding system, SD-Standard deviation.

**Note**: Excluded the patients/Claims having claim amount zero

*Net cost reported is paid by the payer

Numbers are not mutually exclusive

**Table S6** Disease specific healthcare resource utilization and costs (net cost) by activity type for specific events of interest

| **Healthcare Utilization: Number of Visits (Claims)** | | | | | | | | | |
| --- | --- | --- | --- | --- | --- | --- | --- | --- | --- |
| **Event of Interest** | **Cardiac rhythm abnormalities** | | **Hepatic events** | **Diarrhea** | **Neutropenia** | **Venous thromboembolism** | **Heart Failure** | **Ischemia and Infarction** | **Interstitial lung disease** |
| **Overall claims** | | 1720 | 1808 | 1208 | 1326 | 683 | 441 | 45 | 87 |
| **Medications** | | | | | | | | | |
| N (patient counts) | | 231 | 214 | 313 | 220 | 96 | 66 | 18 | 15 |
| Total | | 398 | 349 | 501 | 533 | 252 | 155 | 31 | 31 |
| Mean (SD) | | 1.7 (1.6) | 1.6 (1.4) | 1.6 (1.4) | 2.4 (2.8) | 2.6 (2.9) | 2.3 (2.2) | 1.7 (1.7) | 2.1 (1.6) |
| **CPT Procedures** | |  |  |  |  |  |  |  |  |
| N (patient counts) | | 394 | 470 | 250 | 231 | 108 | 71 | 16 | 17 |
| Total | | 678 | 928 | 369 | 526 | 223 | 145 | 18 | 24 |
| Mean (SD) | | 1.7 (1.5) | 2.0 (1.5) | 1.5 (1.0) | 2.3 (2.3) | 2.1 (2.6) | 2.0 (1.7) | 1.1 (0.3) | 1.4 (0.8) |
| **HCPCS (Consumables)** | | | | | | | | | |
| N (patient counts) | | 21 | 22 | 13 | 26 | 12 | 1 | 7 | - |
| Total | | 23 | 23 | 16 | 29 | 14 | 1 | 8 | - |
| Mean (SD) | | 1.1 (0.4) | 1.0 (0.2) | 1.2 (0.4) | 1.1 (0.3) | 1.2 (0.4) | 1.0 (-) | 1.1 (0.4) | - |
| **Services** | | | | | | | | | |
| N (patient counts) | | 400 | 332 | 251 | 145 | 86 | 65 | 17 | 19 |
| Total | | 617 | 508 | 319 | 236 | 192 | 137 | 23 | 32 |
| Mean (SD) | | 1.5 (1.2) | 1.5 (1.0) | 1.3 (0.7) | 1.6 (1.4) | 2.2 (2.2) | 2.1 (1.7) | 1.4 (0.8) | 1.7 (1.0) |
| **DRG** | | | | | | | | | |
| N (patient counts) | | 4 |  | 2 | 2 | 1 | 2 | 1 | - |
| Total | | 4 |  | 3 | 2 | 2 | 3 | 2 | - |
| Mean (SD) | | 1.0 |  | 1.5 (0.7) | 1.0 |  | 1.5 (0.7) | 2.0 (-) | - |
| **Healthcare Cost (In USD)** | | | | | | | | | |
| **Event of Interest** | | **Cardiac rhythm abnormalities** | **Hepatic events** | **Diarrhea** | **Neutropenia** | **Venous thromboembolism** | **Heart Failure** | **Ischemia and Infarction** | **Interstitial lung disease** |
| **Medications** | | | | | | | | | |
| N (patient counts) | | 231 | 214 | 313 | 220 | 96 | 66 | 18 | 15 |
| Total cost (USD) | | 32466.2 | 252425.7 | 78129.3 | 419341.0 | 57299.0 | 15233.1 | 10252.7 | 847.4 |
| Mean (SD) | | 140.5 (511.7) | 1179.6 (4942.5) | 249.7 (1446.7) | 1906.1 (6661.0) | 596.9 (2118.0) | 230.9 (415.0) | 569.7 (718.6) | 56.4  (80.6) |
| **CPT Procedures** | | | | | | | | | |
| N (patient counts) | | 394 | 470 | 250 | 231 | 108 | 71 | 16 | 17 |
| Total | | 115958.8 | 160959.9 | 38842.7 | 63713.9 | 40139.7 | 35355.6 | 44647.9 | 7636.4 |
| Mean (SD) | | 294.4 (609.7) | 342.6 (677.5) | 155.5 (335.5) | 275.8 (800.8) | 371.7 (511.7) | 498.0 (1015.1 | 2790.5 (4378.1) | 449.3 (385.3) |
| **HCPCS (Consumables)** | | | | | | | | | |
| N (patient counts) | | 21 | 22 | 13 | 26 | 12 | 1 | 7 | - |
| Total cost (USD) | | 2138.4 | 1615.8 | 15189 | 343.4 | 1399.1 | 1.4 | 30878.2 | - |
| Mean (SD) | | 101.8 (165.8) | 73.5 (99.7) | 116.8 (163.4) | 13.3 (13.1) | 116.5 (134.0) | 1.4  (-) | 4411.3  (7370.4) | - |
| **Services** | | | | | | | | | |
| N (patient counts) | | 400 | 332 | 251 | 145 | 86 | 65 | 17 | 19 |
| Total cost (USD) | | 50555.8 | 40434.0 | 26720.7 | 20941.1 | 19715.2 | 12323.3 | 28245.3 | 2850.2 |
| Mean (SD) | | 126.3 (136.2) | 121.7 (107.0) | 106.5 (95.3) | 144.3 (141.1) | 229.3 (298.4) | 189.5 (183.5) | 1661.6 (2,067.9) | 150.0  (120.1) |
| **DRG** | | | | | | | | | |
| N (patient Counts) | | 4 | - | 2 | 2 | 1 | 2 | 1 | - |
| Total cost (USD) | | 20800.0 | - | 17430.0 | 26066.3 | 61788.2 | 11355.2 | 29350.6 | - |
| Mean (SD) | | 5200.1 (2969) | - | 8715.0 (6663.2) | 13033.2 (12734.7) | 61788.2  (-) | 5677.8 (2434.6) | 29350.6  (-) | - |

Source for conversion of AED to USD currency: https://www.unitconverters.net/currency/aed-to-usd.htm; Accessed on 19 April 2023 10:30:00

1 AED (United Arab Emirates Dirham) = 0.2723014922 United States dollar (currency values in USD rounded off to one decimal point)

CPT-Current procedural terminology, DRG-Diagnosis related group, HCPCS-Healthcare common procedure coding system, SD-Standard deviation.

**Note**: Excluded the patients/Claims having claim amount zero

*Net cost reported is paid by the payer

Numbers are not mutually exclusive

**Table S7** Events of special interest among CDK4/6 inhibitor treated patients during post-index period

|  | **Overall** | | **Overall %** |
| --- | --- | --- | --- |
| **Overall study population in index period** | **8031** | | **100.0%** |
| Overall study population in post-index period | 7698 | | 95.9% |
| Overall CDK4/6 patients in the post-index period | 174 | | 2.3% |
| Overall event of special interest patients in study period and who are being treated with CDK4/6 inhibitors in the Post-Index Period | 116 | | 66.7% |
| Continuous enrollment: Overall CDK4/6 inhibitors during the 12‑months post-index follow-up period and having at least one claim in the 12-months post-index follow-up period (from CDK4/6 Index date to Index date +365 days) | 94 | | 81.0% |
| Overall event of special interest patients who are being treated with CDK4/6 inhibitors during the 12-months post-index follow-up period (from CDK4/6 Index date to Index date +365 days) | 73 | | 77.7% |
| **Event of special interest** | **Number of patients** | **% of patients** | **Cumulative % of patients** |
| Cardiac rhythm abnormalities^a^ | 23 | 19.8% | 19.8% |
| Hepatic events | 24 | 20.7% | 40.5% |
| Diarrhea | 26 | 22.4% | 62.9% |
| Neutropenia | 35 | 30.2% | 93.1% |
| Venous thromboembolism | 5 | 4.3% | 97.4% |
| Heart failure | 1 | 0.9% | 98.3% |
| Interstitial lung disease | 2 | 1.7% | 100.0% |

CDK-Cyclin-dependent kinase, HCRU-Healthcare resource utilization, ICD-10 CM-International classification of diseases 10^th^ revision clinical modification.

^a^ Cardiac rhythm abnormalities group included abnormalities of heart rhythm, arrhythmia, atrial fibrillation and flutter, cardiac arrhythmias, and heart block in this study.

**Appendix**

**ICD-10 codes for diagnosis of breast cancer**

| **ICD-10 CM codes** | **Diagnosis Name** | **Type** |
| --- | --- | --- |
| C50.011 | Malignant neoplasm of nipple and areola, right female breast | Female |
| C50.012 | Malignant neoplasm of nipple and areola, left female breast | Female |
| C50.019 | Malignant neoplasm of nipple and areola, unsp female breast | Female |
| C50.111 | Malignant neoplasm of central portion of right female breast | Female |
| C50.112 | Malignant neoplasm of central portion of left female breast | Female |
| C50.119 | Malignant neoplasm of central portion of unsp female breast | Female |
| C50.211 | Malig neoplm of upper-inner quadrant of right female breast | Female |
| C50.212 | Malig neoplasm of upper-inner quadrant of left female breast | Female |
| C50.219 | Malig neoplasm of upper-inner quadrant of unsp female breast | Female |
| C50.311 | Malig neoplm of lower-inner quadrant of right female breast | Female |
| C50.312 | Malig neoplasm of lower-inner quadrant of left female breast | Female |
| C50.319 | Malig neoplasm of lower-inner quadrant of unsp female breast | Female |
| C50.411 | Malig neoplm of upper-outer quadrant of right female breast | Female |
| C50.412 | Malig neoplasm of upper-outer quadrant of left female breast | Female |
| C50.419 | Malig neoplasm of upper-outer quadrant of unsp female breast | Female |
| C50.511 | Malig neoplm of lower-outer quadrant of right female breast | Female |
| C50.512 | Malig neoplasm of lower-outer quadrant of left female breast | Female |
| C50.519 | Malig neoplasm of lower-outer quadrant of unsp female breast | Female |
| C50.611 | Malignant neoplasm of axillary tail of right female breast | Female |
| C50.612 | Malignant neoplasm of axillary tail of left female breast | Female |
| C50.619 | Malignant neoplasm of axillary tail of unsp female breast | Female |
| C50.811 | Malignant neoplasm of ovrlp sites of right female breast | Female |
| C50.812 | Malignant neoplasm of ovrlp sites of left female breast | Female |
| C50.819 | Malignant neoplasm of ovrlp sites of unsp female breast | Female |
| C50.911 | Malignant neoplasm of unsp site of right female breast | Female |
| C50.912 | Malignant neoplasm of unspecified site of left female breast | Female |
| C50.919 | Malignant neoplasm of unsp site of unspecified female breast | Female |
